# Supplementary material for: Circulating immunome fingerprint in eosinophilic esophagitis is associated with clinical response to proton pump inhibitor treatment
Source: Front Immunol. 2024 Apr 5;15:1374611. doi: 10.3389/fimmu.2024.1374611 (PMC11026586; doi:10.3389/fimmu.2024.1374611)
Supplement: Supplementary file 1 [file DataSheet_1.pdf]

Supplementary Figure 1

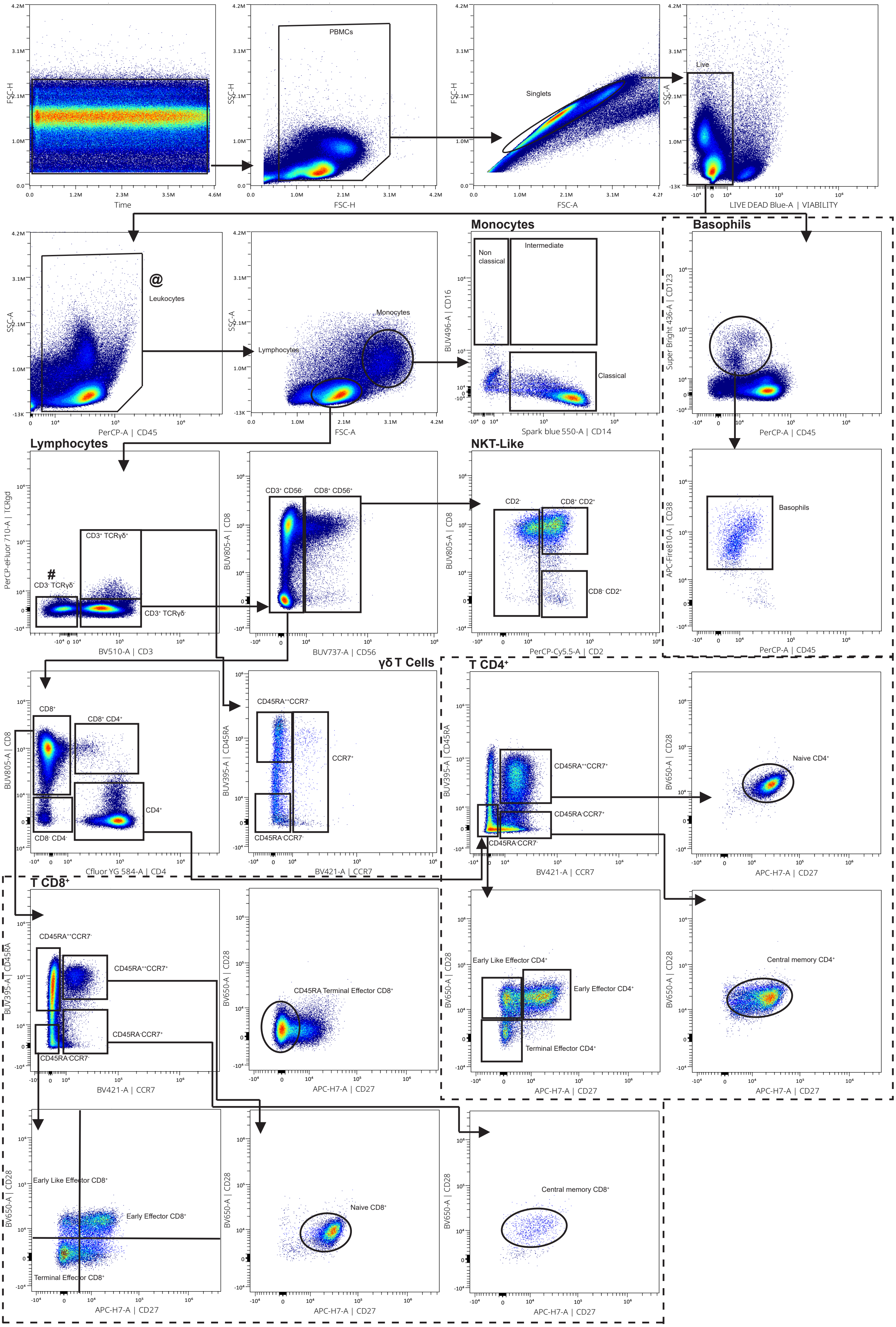

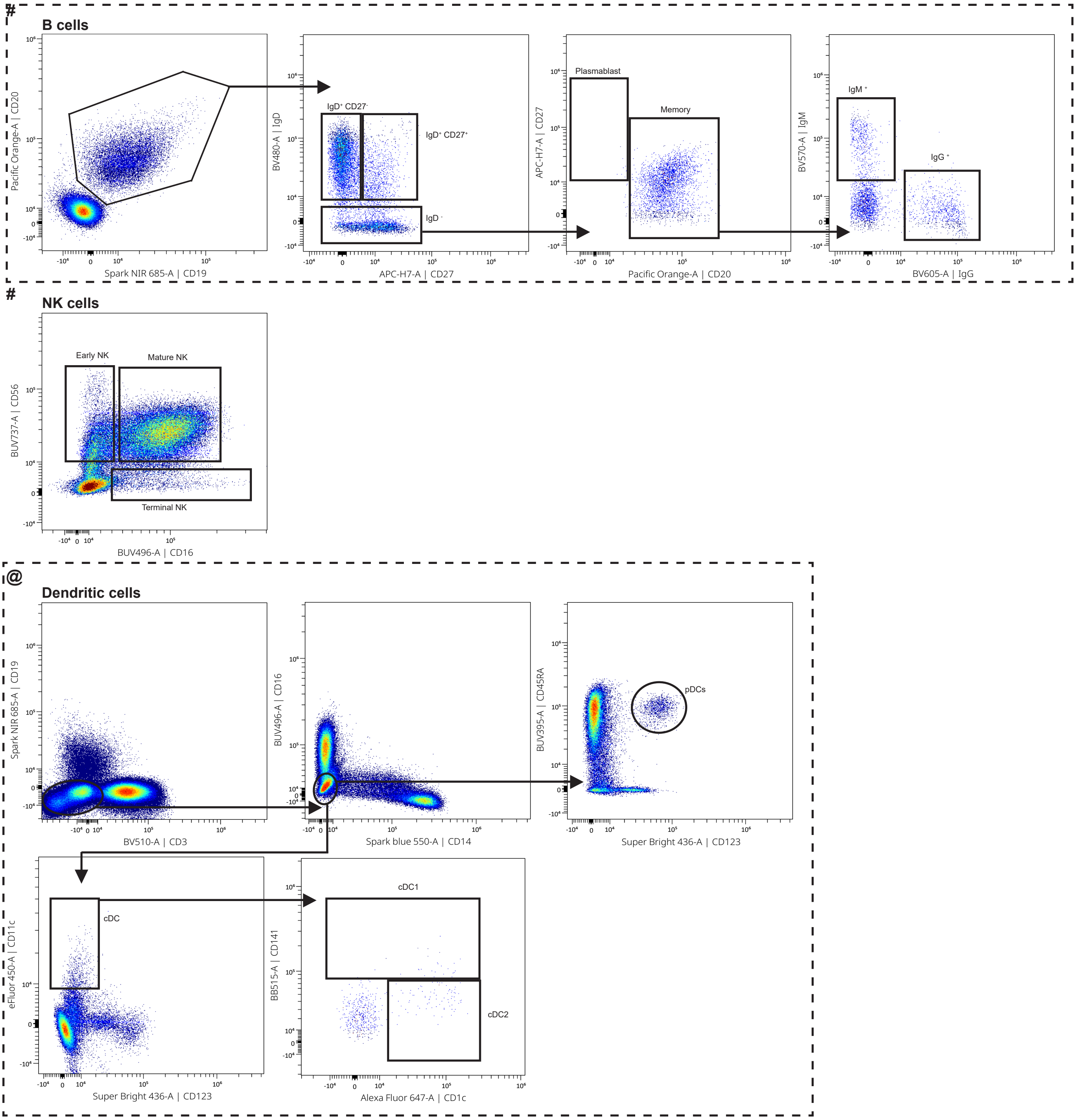

Supplementary Figure 1. Hierarchical gating strategy for cluster classification.
Representative gating strategy used to identify within total peripheral blood mononuclear cells (PBMC) the main leukocyte populations. Arrows and gates are used to visualize the relationships across plots. After doublets and dead cells were excluded, basophils were identified as $CD45^{+}CD123^{+}CD38^{+}$. Monocytes were gated based on FSC-A/SSC-A properties and further classified into non-classical ($CD14^{-}CD16^{+}$), intermediate ($CD14^{+}CD16^{+}$/low), and classical ($CD14^{+}CD16^{-}$). From the lymphogate, T-cell were identified based on the expression of CD3. Total Tγδ cells were identified as $CD3^{+}TCR\gamma\delta^{+}$ and subdivided based on the expression of CD45RA and CCR7. Total NKT-like cells were identified in the $CD3^{+}TCR\gamma\delta^{-}$ compartment as $CD56^{+}$. The inclusion of CD2 and CD8 further classified the NKT-like cells. Total T-cells were defined as $CD3^{+}TCR\gamma\delta^{-}CD56^{-}$ and further divided into $CD4^{+}$, $CD8^{+}$, $CD4^{+}CD8^{+}$ and $CD4^{-}CD8^{-}$ T-cells. Within total $CD4^{+}$ T-cells and $CD8^{+}$ T-cells, CCR7, CD45RA, CD27, and CD28 were further used to divide them into different T-cell phenotypes. B-cells were gated from the $CD3^{-}TCR\gamma\delta^{-}$ as $CD19^{+}$ and/or $CD20^{+}$ cells. B-cells were further classified as $IgD^{+}CD27^{-}$, $IgD^{+}CD27^{+}$, or $IgD^{-}CD27^{+/-}$; the $IgD^{-}CD27^{+/-}$ subset was divided into plasmablasts or IgD^{-} memory B cells based on CD20 expression and CD27. Memory cells were classified in IgM^{+} or IgG^{+}. NK cells were defined within the lymphogate as $CD3^{-}TCR\gamma\delta^{-}$ and classified as early NK ($CD56^{+}CD16^{-}$), mature NK ($CD56^{+}CD16^{+}$), and terminal NK ($CD56^{-}CD16^{+}$) cells. Dendritic cells were identified within $CD3^{-}CD19^{-}$ as $CD14^{-}CD16^{-}$. Further gating with $CD123^{+}CD45RA^{+}$ determined plasmacytoid DCs, (pDC) and $CD123^{-}CD11c^{+}$ as classical or conventional DC (cDC). cDC were divided into type 1 ($CD141^{+}$, cDC1) and type 2 ($CD1c^{+}$, cDC2).

Supplementary Figure 2

C-56 Central Memory CD4⁺ (1)

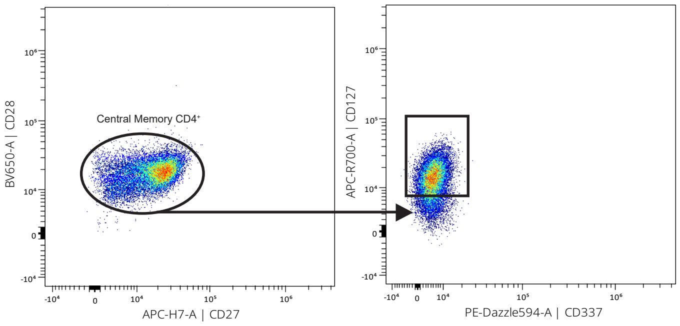

C-69 Early Effector CD8⁺

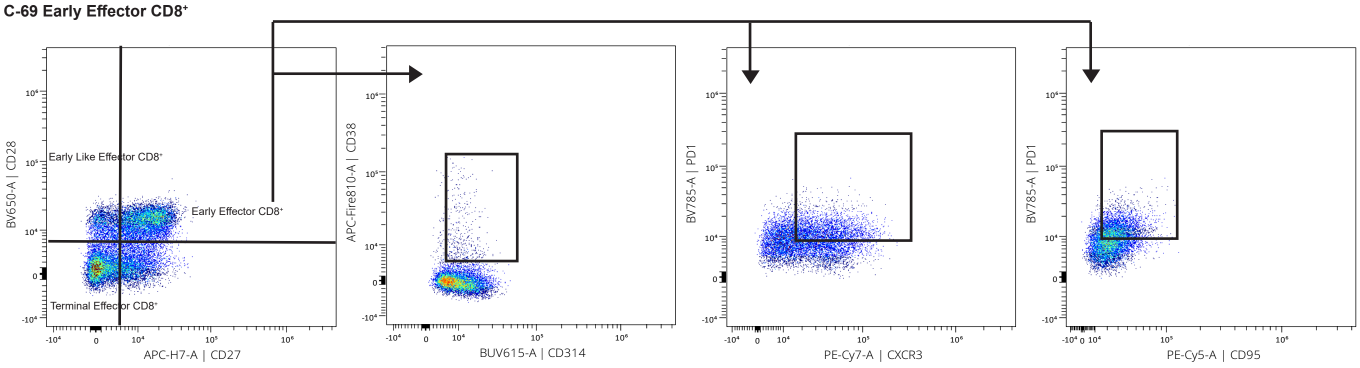

C-37 CD4⁻ CD8⁻

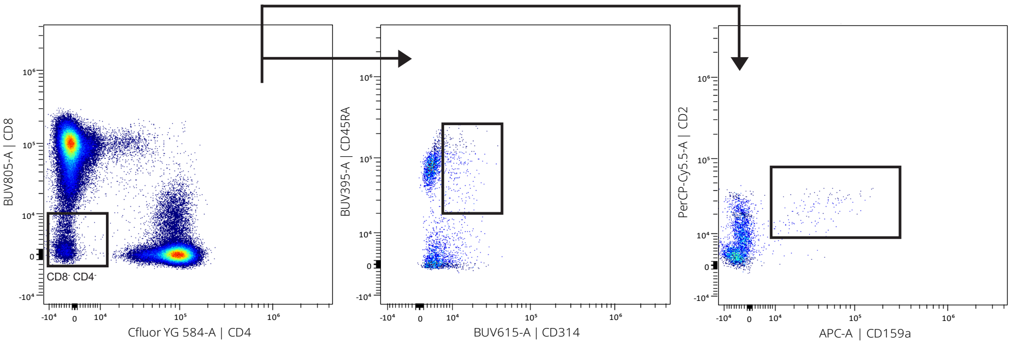

C-4 NK Mature (4)

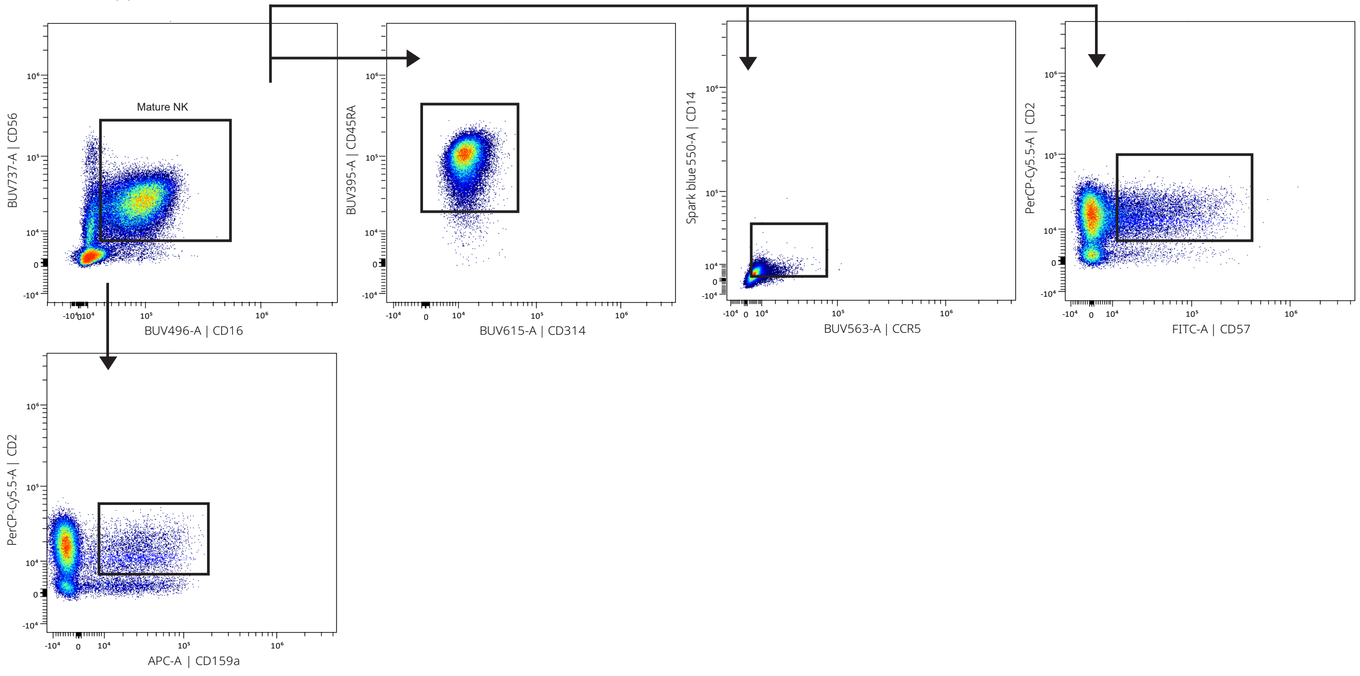

C-22 No Memory $CD27^-$

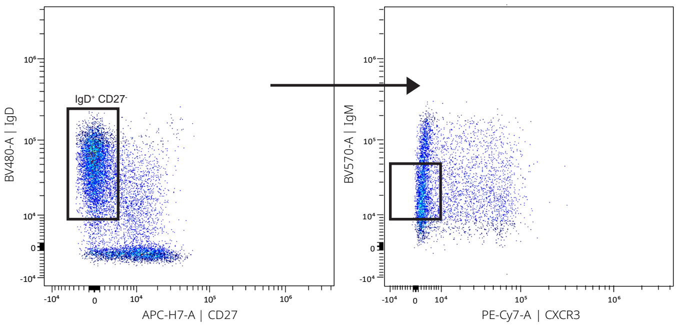

C-26 Denditic cells pDC

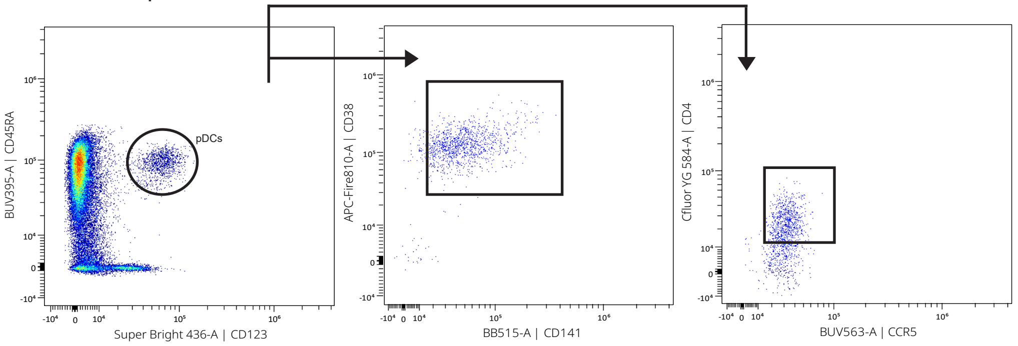

C-27 Monocytes Classical (1)

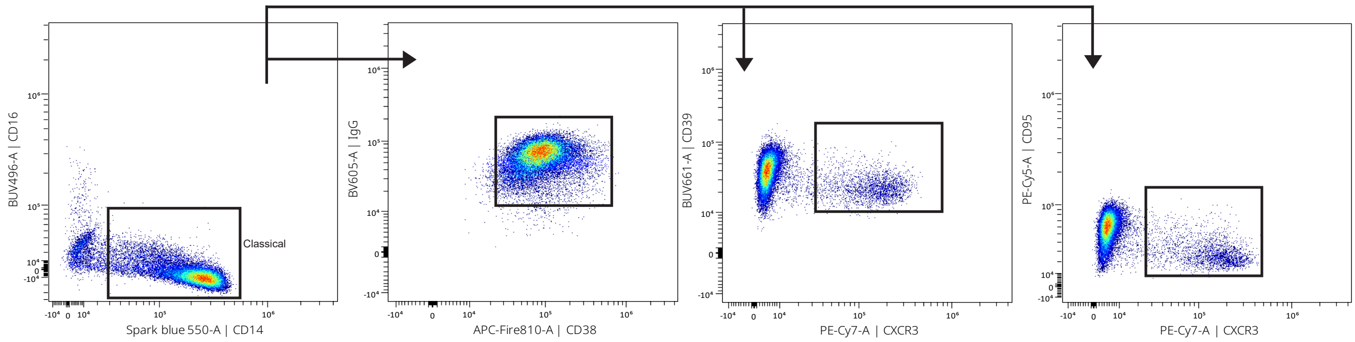

C-33 Unknown Mieloid APC

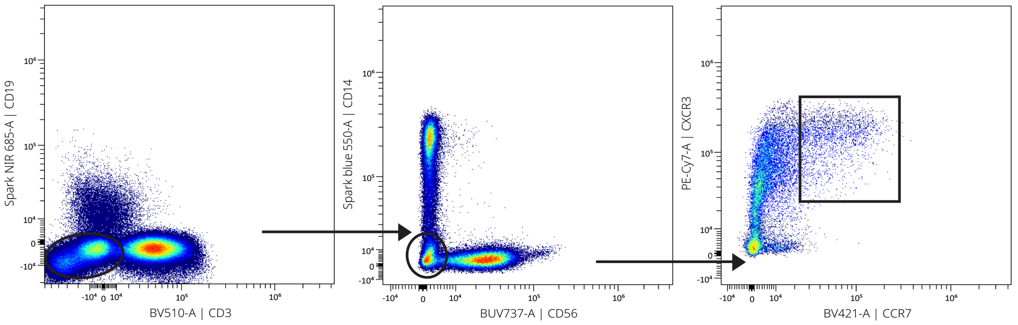

C-14 Basophil

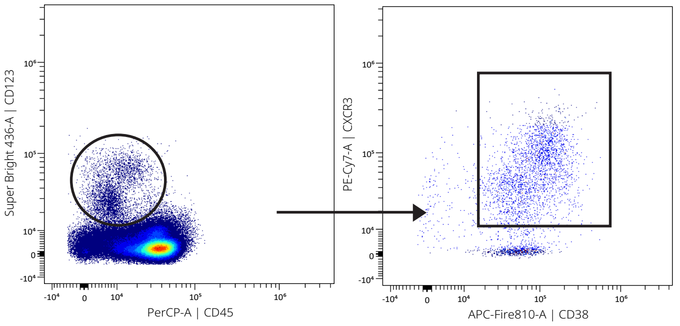

Supplementary Figure 2. Gating strategy for significant cluster validation.
Cell clusters identified in Table 1 which were statistically significant found among the different comparisons performed were validated further identified following classical hierarchical gating approaches. C-56 was gated from central memory CD4 $CD127^+$. C-69 was identified from early effector CD8 which were $CD38^+$ $CD314^+$ $PD1^+$ $CXCR3^+CD95^+$. C-37 was classified from $CD8^-CD4^-$ as $CD314^+$ $CD45RA^+$ $CD2^+$ $CD159a^+$. C-4 subset was identified as Mature NK $CD45RA^+$ $CD314^+$ $CD14^+$ $CCR5^+$ $CD2^+$ $CD57^+$ $CD159a^+$. C-22 was gated from No Memory $CD27^-$ with IgM^+ $CXCR3^-$. C-26 was defined as $CD38^+$ $CD141^+$ $CD4^+$ $CCR5^+$. C-27 were classified from classical monocytes IgG^+ $CD38^+$ $CD39^+$ $CXCR3^+$ $CD95^+$. C-33 was gated from CD19- $CD3^-$ $CD14^-$ $CD56^-$ $CXCR3^+$ $CCR7^+$. C-14 was defined as basophil $CXCR3^+$ $CD38^+$.

Supplementary Figure 3

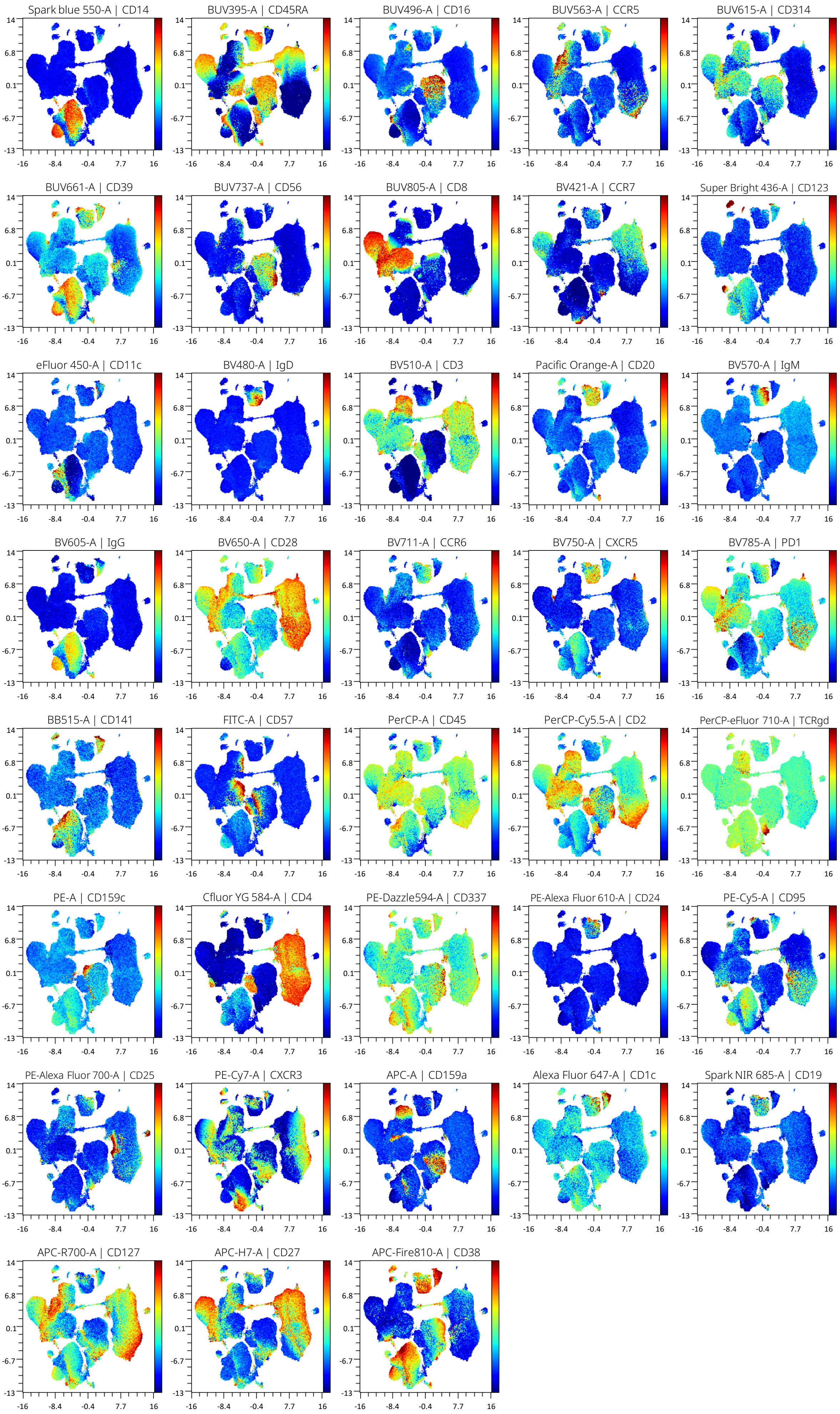

Supplementary Figure 3. Marker expression on the UMAP from peripheral blood mononuclear cells. Surface expression intensities of all 37 analysed markers is shown by a colour code based on the intensity. Red represents higher expression and blue represents lower expression.

Supplementary Figure 4

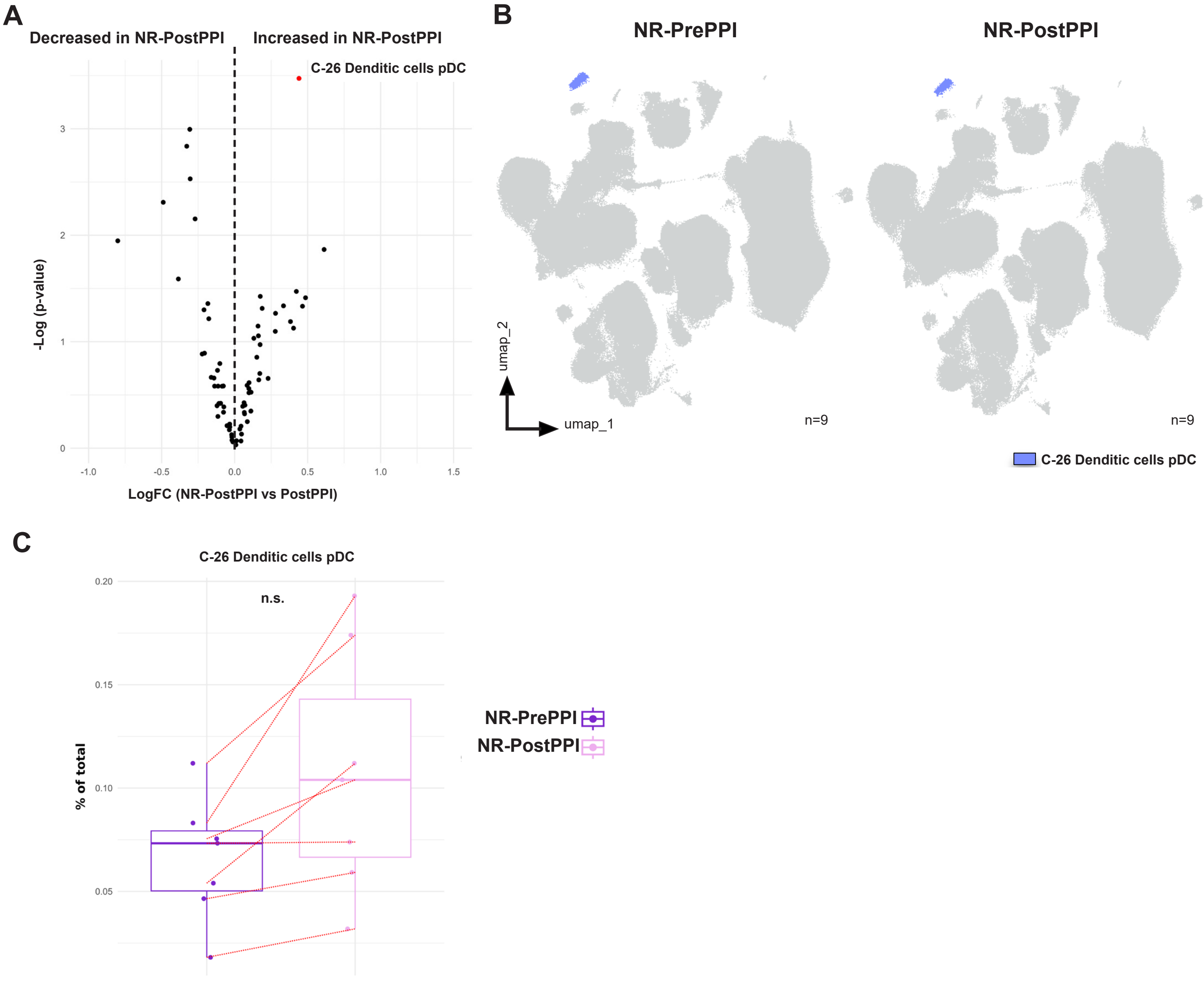

Supplementary Figure 4. Non-Responders do not recover pDC values after treatment.
(A) Volcano plot of differential analysis between NR-PrePPI (n=9) and NR-PostPPI (n=9). LogFC and -Log(p-value) is represented. (B) UMAP representation from NR-PrePPI and PostPPI patients with MC-26 coloured. (C) Validation by classic gating of MC-26 as shown in Supplementary Figure 1 and 2. Boxplot with MC-26 where individual percent of total value, group median and minimum and maximum values is represented. Red line is indicating paired samples PrePPI and PostPPI. Statistical significance was established by paired t-test analysis considering a p-value <0.05 as statistically significant (*p<0.05) (n=7). Non-Responder (NR), before treatment (PrePPI), after treatment (PostPPI).

Supplementary Table 1

Specificity	Fluorochrome	Clone	Provider
CCR5	BUV563	2D7/CCR5	BD Biosciences
CCR6	BV711	G034E3	BioLegend
CCR7	BV421	G043H7	BioLegend
CD1c	Alexa Fluor 647	L161	BioLegend
CD2	PerCP-Cy5.5	TS1/8	BioLegend
CD3	BV510	SK7	BioLegend
CD4	cFluor YG584	SK3	CYTEK
CD8	BUV805	SK1	BD Biosciences
CD11c	eFluor 450	3,9	Thermo Fisher
CD14	Spark Blue 550	63D3	BioLegend
CD16	BUV496	3G8	BD Biosciences
CD19	Spark NIR 685	HIB19	BioLegend
CD20	Pacific Orange	HI47	Thermo Fisher
CD24	PE-Alexa Fluor 610	SN3	Thermo Fisher
CD25	PE-Alexa Fluor 700	CD25-3G10	Thermo Fisher
CD27	APC-H7	M-T271	BD Biosciences
CD28	BV650	CD28.2	BioLegend
CD38	APC-Fire 810	HIT2	BioLegend
CD39	BUV661	TU66	BD Biosciences
CD45	PerCP	2D1	BioLegend
CD45RA	BUV395	5H9	BD Biosciences
CD56	BUV737	NCAM16.2	BD Biosciences
CD57	FITC	HNK-1	BioLegend
CD95	PE-Cy5	DX2	BioLegend
CD123	Super Bright 436	6H6	Thermo Fisher
CD127	APC-R700	HIL-7R-M21	BD Biosciences
CD141	BB515	1A4	BD Biosciences
CD159a	APC	REA110	Miltenyi
CD159c	PE	REA205	Miltenyi
CD314	BUV615	1D11	BD Biosciences
CD337	PE-Dazzle 594	P30-15	BioLegend
CXCR3	PE-Cy7	G025H7	BioLegend
CXCR5	BV750	RF8B2	BD Biosciences
IgD	BV480	IA6-2	BD Biosciences
IgG	BV605	G18-145	BD Biosciences
IgM	BV570	MHM-88	BioLegend
PD-1	BV785	EH12.2H7	BioLegend
TCRγδ	PerCP-eFluor 710	B1.1	Thermo Fisher
Viability	Live Dead Blue-A	-	Thermo Fisher

Supplementary Table 1.
Specificity, fluorochrome, clone and provider of the different antibodies used.

Supplementary Table 2

C	Population	Subset	Phenotypic expression	Functional expression
1	NK	Mature (1)	**CD16** CD56 CD45RA	CD159c **CXCR3** CD314
2	NK	Mature (2)	**CD16** CD56 CD45RA	**CD2 CD57** CD159c **CXCR3** CD314
3	NK	Mature (3)	**CD16** CD56 CD45RA	CD2 CD57 CD159c CD314
4	NK	Mature (4)	**CD16** CD56 CD45RA	CD314 **CD14 CCR5 CD2 CD159a CD57**
5	NK	Mature (5)	**CD16** CD56 CD45RA CD38 CD8	**CD2** CD314
6	NK	Mature (6)	**CD16** CD56 CD45RA	**CD2** CD314
7	NK	Mature (7)	**CD16** CD56 CD45RA CD38	CD314
8	NK	Early to Mature	**CD16dim** CD56 CD45RA^{++}	CD2 CD314 CD159a
9	NK	Early (1)	CD45RA CD56 CD38	CD314 CD337
10	NK	Early (2)	CD45RA CD56 CD38	CD314 **CD159a** CD337 **CD2**
11	NK	Early (3)	CD45RA CD56 CD38	**CXCR3**
12	NK	Early (4)	CD45RA CD56	
13	Unknown		CD45RA CD25 CD127	
14	Basophil		CD123 CD38	CXCR3
15	B	Memory	CD20 CD19 CD45RA CD27dim CD38	CD39 CCR7 CCR6 CXCR5 CD95 CD1c
16	B	Memory IgG^{+}	CD20 CD19 CD45RA^{+} **IgG** CD38	CCR6 CXCR5 CD159c CD337
17	B	Memory IgM^{+} (1)	CD20 CD19 CD45RA^{++} **IgM** CD24 CD25 CD38	CCR7 **CCR5** CCR6 CXCR5 PD1 **CD337** CD1c
18	B	Memory IgM+ (2)	CD20 CD19 CD45RA^{++} **IgM** CD24 CD25 CD38	CCR7 CCR6 CXCR5 PD1 CD1c
19	B	No Memory CD27^{-} (1)	CD20 CD45RA CD38	CCR7 CCR6 CXCR5 **CXCR3**
20	B	No Memory CD27^{-} (2)	CD20 CD19 CD45RA^{++} **IgD** CD38	CCR7 CCR6 CXCR5 **CXCR3**
21	B	No Memory CD27^{-} (3)	CD20 CD19 CD45RA^{++} **IgD^{++} IgMdim** CD38	CCR7 CCR6 CXCR5 **PD1**
22	B	No Memory CD27^{-} (4)	CD20 CD19 CD45RA IgD CD38	CCR7 CCR6 CXCR5
23	Dendritic cells	cDC2 (1)	**CD11c** CD141 CD38 CD1c	CD39 CCR5 CD95
24	Dendritic cells	cDC2 (2)	**CD11c** CD141 CD38 CD1c	CD39 CCR5 CD95 CXCR3
25	Dendritic cells	cDC1	**CD11c** CD141 CD38 CD1c	CD39 CCR5 CD95 CXCR3
26	Dendritic cells	pDC	**CD123 CD45RA** CD141lo CD38 CD4	CCR5 CXCR3
27	Monocytes	Classical (1)	**CD14^{++}** IgG CD38	CD39 **CXCR3** CD95
28	Monocytes	Classical (2)	**CD14** CD45RA CD11c IgG CD141 CD38	CD95 CD39
29	Monocytes	Classical (3)	**CD14** IgG CD38	CD95 CD39
30	Monocyte	Classical (4)	CD14 CD39 IgG CD95	
31	Monocytes	Non-classical	**CD16** CD45RA CD11c CD141 CD38	
32	Unknown	myeloid antigen presenting cells (1)	CD45RAdim CD11c CD38	
33	Unknown	myeloid antigen presenting cells (2)	CD123 CD11c CD141 CD38	**CXCR3**
34	NKT-Like cells	CD2^{+} CD8^{+}	CD3 CD4 **CD8dim CD2** CD45RA CD56	CD57
35	Tcell	CD4^{+} CD8^{+}	CD3 **CD4 CD8** CD28 CD27 CD25	CD2 CD95
36	Tcell	CD4^{-} CD8^{-} (1)	CD3 CD28 CD27dim	CD314 **CCR5** CD2 **CD159a CXCR3**
37	Tcell	CD4^{-} CD8^{-} (2)	CD3 CD45RA	CD314 CD2 CD159a
38	Tcell	CD4^{-} CD8^{-} (3)	CD3 CD45RA CD27dim	CD314 CD2
39	Tcell	CD4^{-} CD8^{-} (4)	CD3 CD28 CD27dim	CD314 **CCR5** CD2 **CXCR3**
40	Tcell	CD4^{-} CD8^{-} (5)	CD3 CD28	**CCR5** CD2
41	Tcell	CD4^{-} CD8^{-} (6)	CD3 CD28dim CD27dim	CCR5 **CXCR3**
42	Tcell	CD4^{-} CD8^{-} (7)	CD3 CD45RA CD27 CD28	CXCR3 **CCR7**
43	Tcell	γδ CD45RA^{+}/CCR7^{-}	CD3 **TCRγδ CD45RA** CD141 CD25 CD1c	CD314 CCR6 CD2
44	Tcell	CD4 Terminal Effector CD45RA like (1)	CD3 CD4 **CD45RA** CD56dim	CD2
45	Tcell	CD4 Terminal Effector CD45RA like (2)	CD3 CD4 **CD45RA**	CD2 **CD57**
46	T cell	Terminal effector CD4 (1)	CD3 CD4	CD2 PD1 **CD57**
47	T cell	Terminal effector CD4 (2)	CD3 CD4	CD2 PD1
48	Tcell	CD4 Naive (1)	CD3 CD4 **CD45RA^{++} CCR7** CD28 CD27	
49	Tcell	CD4 Naive (2)	CD3 CD4 **CD45RA CCR7** CD28 CD27	
50	Tcell	CD4 Naive (3)	CD3 CD4 **CD45RA CCR7** CD28 CD27	CXCR3
51	Tcell	CD4 Naive (4)	CD3 CD4 **CD45RA^{++} CCR7** CD28 CD27	CXCR3
52	Tcell	Early like Effector CD4	CD3 CD4 **CD28** CD127 CD25lo	CD2 CD95
53	Tcell	Early Effector CD4 (1)	CD3 CD4 **CD28 CD27** CD127	CD2 **CXCR3** CD95
54	Tcell	Early Effector CD4 (2)	CD3 CD4 **CD28 CD27** CD127	**CD39dim** CD2 CD95
55	Tcell	Early Effector CD4 (3)	CD3 CD4 **CD28 CD27** CD25	CD95
56	Tcell	Central Memory CD4 (1)	CD3 CD4 **CCR7 CD28 CD27** CD127	CD337 CD95
57	Tcell	Central Memory CD4 (2)	CD3 CD4 **CCR7 CD28 CD27** CD127	**CD337 CXCR3** CD95
58	Tcell	Central Memory CD4 (3)	CD3 CD4 **CCR7 CD28 CD27**	**CXCR3** CD95
59	Tcell	CD8 Naive (1)	CD3 CD8 **CD45RA CCR7 CD27**	CD314 **CXCR3**
60	Tcell	CD8 Naive (2)	CD3 CD8 **CD45RA CCR7 CD27**	CD314
61	Tcell	Terminal Effector CD45RA CD8 (1)	CD3 CD8 **CD45RA**	CD314
62	Tcell	Terminal Effector CD45RA CD8 (2)	CD3 CD8 **CD45RA**	CD314 **CD2**
63	Tcell	Terminal Effector CD45RA CD8 (3)	CD3 CD8 **CD45RA**	CD314 **CD2 CD57**
64	Tcell	Terminal Effector CD45RA CD8 (4)	CD3 CD8 **CD45RA**	CD314 **CD2 CXCR3**
65	Tcell	Terminal Effector CD8 (1)	CD3 CD8	CD314 CD2 **CD57**
66	Tcell	Terminal Effector CD8 (2)	CD3 CD8	CD314 CD2 **PD1**
67	Tcell	Early Effector CD8 (1)	CD3 CD8 CD45RAlo **CD28 CD27** CD127	CD314 CD2 PD1
68	Tcell	Early Effector CD8 (2)	CD3 CD8 **CD28 CD27**	CD314 **CXCR3**
69	Tcell	Early Effector CD8 (3)	CD3 CD8 CD28 CD27 CD38	CD314 PD1 CXCR3 CD95
70	Tcell	Early Effector CD8 (4)	CD3 CD8 **CD28 CD27** CD127	CD314 **CCR5 CD2 CXCR3 CD95 PD1 CD159a**
71	Tcell	Early Effector CD8 (5)	CD3 CD8 **CD28 CD27** CD127	CD314 **CD2 PD1 CXCR3 CD95**
72	Tcell	Early Effector CD8 (6)	CD3 CD8 **CD28 CD27** CD127 CD2	CD314 **CCR5 PD1**
73	Tcell	Early like Effector CD8	CD3 CD8 **CD28** CD127	CD314 **CCR5 CD2**

Supplementary Table 2. Cell cluster identification from control and EoE patients.
For each of the 73 identified FlowSOM clusters its ontogeny and subset is shown, together with the specific subset, phenotype and expression of functional markers. Markers highlighted in bold denote differential expression within the same population.
